# Weathering violence: Atmospheric materialities and olfactory durations of ‘skunk water’ in Palestine

**DOI:** 10.1177/25148486241226923

**Published:** 2024-01-30

**Authors:** Mikko Joronen, Wassim Ghantous

**Affiliations:** 7840 Tampere University, Finland; 7840 Tampere University, Finland

**Keywords:** Materiality, weathering, meteorology, atmospheres, skunk water, olfactory violence, duration, coloniality of smell, Palestine/Israel, crowd control technologies

## Abstract

This paper examines a particular technique of weaponising smell – the spraying of ‘skunk water’, a crowd control tool originally developed by the Israeli Police to disperse Palestinian protests – and the olfactory atmospheres of moving matter it extends its violence through. It focuses particularly on ways in which skunk water spraying operates by ‘weathering’ the air with a stench that sticks on bodies, objects and spaces, often for considerably long periods. By elaborating the two entwined aspects of weathering – the weaponising and the meteorological – the paper shows how skunk water spraying engenders malodourous olfactory durations that move and through their movement extend their violence through meteorological fluidities and moving bodies/objects. The violence of skunk water, we so argue, contains lingering tempos that through material morphoses (water, mist, droplets, dried powder), reactivating/intensifying weather conditions (rain, heat, humidity, wind), and material kinetics (moving bodies, objects and air) spatialise proximities of atmospheric stench, hence targeting the way breathing bodies are immersed in their olfactory environments. By comprehending weathering as weaponised ‘matter in motion’, the paper offers a novel way of thinking about atmospheric violence through non-linear movements and lingering proximities – namely, as a weaponisation of an olfactory duration of a stinky matter that moves.

## Introduction

This paper examines the use of a particular atmospheric technology of policing – the ‘skunk water’ – and the materialities it is constituted through. Co-developed by the Israeli Police and Odortec company in 2004, skunk water was designed to quell Palestinian demonstrations and gatherings through spraying of extremely foul-smelling liquid most commonly fired from water cannons placed at the top of armoured vehicles called in Arabic, humorously, the *Kharrarah* (shit-spreader) (on recent use of drones and boats, see Eisenberg, 2018; Mahmoud and Shehada, 2019; UNHRC, 2019). Promoted as an ‘eco-friendly’, ‘organic’, ‘effective’, and ‘non-lethal’ alternative to potentially lethal crowd control tools, skunk water has received global attention and has now been bought, for instance, by police departments in the US (see iHLS, 2018; Khalek, 2015; Lee, 2018). Beyond such brandings, however, skunk water indiscriminately targets people, covers vast areas, and is purposely sprayed to wet entire Palestinian neighbourhoods (homes, shops, religious sites, streets, gardens etc.) with a stench that can cause nausea, gagging, vomiting, and various skin and eye reactions, but importantly also sticks to bodies, objects and surfaces it lands on, often for considerably long time (from days up to several months). The smell, described as a mixture of sewage and rotting corpse, further spreads beyond the areas of spraying, for instance through wind, car tires, or by-passers shoes, while also stretching over time, for instance, when becoming re-activated by specific weather conditions or drying to cracks in buildings. Accordingly, the effects of skunk water on everyday life are as much economic and political as they are social, bodily and affective.

Despite the variety of effects involved in skunk water use – the sensory assaults, financial losses, humiliation, othering of targeted communities, etc. – in this paper we focus particularly on how skunk stench operates as a *duration of moving matter*. It is the lingering duration of skunk stench that, we argue, offers a key for understanding ways in which the movement, even chaotic turbulences of smell are *weathered* as material modalities of colonial violence. By focusing particularly on the weaponisation of meteorological fluidities, we show how the moving materialities of skunk water are ‘weathered’ to engender atmospheres of olfactory violence that, instead of killing or maiming (Puar, 2017), target the way breathing bodies are immersed in their environs (Sloterdijk, 2009). While so approaching skunk water as a material technology of weaponising aerial lingering(s) and moving durations of smell, the violent effects of skunk water spraying, we argue, cannot be separated from its operational logic. Our focus on skunk water as a ‘matter in motion’ (Nail, 2019) thus aims to provide new insights into debates around material and environmental aspects of atmospheric violence (see Adey et al., 2013; Fregonese, 2017; Joronen, 2023; Lambert, 2017; Nieuwenhuis, 2016), particularly on questions related to ways in which atmospheric materiality operates through the olfactory weaponisation of lingering effluvia (in Palestine, and beyond).

Within the existing geographical literature, the notion of smell has been approached from sporadic angles, ranging from scent technologies (Kitson and McHugh, 2019) and art gallery spaces (Straughan, 2015) to geographies of waste infrastructure (Chan, 2020; Stamatopoulou-Robbins, 2022), albeit most commonly olfaction has been acknowledged by incorporating it to more generic references on ‘multisensory’ experiences, encounters, and engagements with one's surroundings (eg. Bell, 2019; Gökariksel and Secor, 2022; Pyyry, 2019). The materiality of olfactory atmospheres, however, has gained surprisingly little attention, considering the amount of work done on cultural and political geographies of atmospheres, affects and materiality (eg. Bissell, 2022; Closs Stephens et al., 2017; Fregonese and Laketa, 2022; Hitchen, 2021; Lorimer et al., 2019; cf. Ojani, 2022; Nieuwenhuis, 2016; Savelli et al., 2022). In a similar vein, prevalent literature on olfaction, policing, and warfare, though focusing on a variety of aspects such as racist representations of the ‘smelly Other’ (Callow, 2017), neurological links between the smell and the war traumas (Woodward et al., 2013), weaponisation of animals’ smelling capabilities (such as the ‘sniffing dogs’), and development of sniffing machines for detection and tracking purposes (McSorley, 2020), has been less keen to look at olfaction as a material technique of atmospheric violence. The olfactory weaponisation of skunk water, we thereby suggest, should be approached as atmospheric in a profoundly material and spatial sense: it operates by weaponising olfactory materialities through meteorological turbulences of ‘here and now’ (Serres, 2018) – through the *weather* – and by so producing its violent effects through the non-linear rhythms of moving and lingering air that the body, as a breathing entity, is fundamentally reliant on.

To grasp these meteorological movements and lingering durations of smell the paper focuses on what we name as the *weathering* of violence. Weathering, we argue, carries a double meaning. Firstly, it signifies a way in which skunk water operates as a political technique that *weaponises* olfactory *durations* by temporalising, spatialising and aerialising – by weathering – the stench through the bodies, objects, surfaces, and air that the skunk water spraying contaminates. With weathering, we hence refer to a process of manufacturing olfactory atmospheres that (mal)odourise the air, bodies, and solid environs, and by doing so, expose sniffing and breathing bodies to lingering, adhesive and moving stink. Secondly, weathering names further the way certain *meteorological* conditions, and the material movements of air they instigate, constantly weather the stench. Here the lingering malodorous of skunk smell evolve through aerial fluidities and particular weather conditions, such as humidity, temperature, and air pressure, that can intensify, move and/or reactivate the skunk stench. The lingering stench thus has a material duration that is not only designed to stick but also moves and lingers due to prevalent weather conditions so mobilised as material mediums of olfactory violence and its various (disciplining, punishing, marking, humiliating and dispossessing) functions. By considering these two aspects together, our discussion of ‘weaponised meteorologies of weathering’ offers a materiality-focused addition to debates elaborating political and colonial violence as a non-linear modality of weaponising chaotic conditions, ungovernability, and unpredictable uncertainties (eg. Davies et al., 2017; Joronen and Griffiths, 2022; Kotef and Amir, 2011; Peteet, 2017; Weizman, 2007), thus expanding the recent, still relatively diminutive and scattered work on geographies of weathering (eg. Collins, 2020; de Vet and Head, 2020; McCormack, 2018; Walker, 2023; in cognate disciplines, see Holzheye and Wedemayer, 2020; Neimanis and Walker, 2014; Sharpe, 2016).

We start the paper by elaborating on how the weaponisation of skunk water (mal)odours functions through the lingering duration of smell. In the second section, we show further how the meteorological motions are a crucial part of how such weaponised weathering operates. Olfactory atmospheres of skunk water are not only place-bound environs of sphereological ‘bubbles’ (Sloterdijk, 2009, 2011) at best transformed by the external forces of movement; *movement*, we rather argue by following the work of Michel Serres (1995; 2018), should be comprehended as a constitutive condition through which transformations, equilibriums, rhythms and turbulences maintain and organise ways in which olfactory violence weathers its lingering proximities. In the third section, which contains two subsections, we focus further on atmospheric weathering, particularly how it operates and produces various modes of olfactory violence in several contexts of skunk water use in Palestine. We aim to approach the moving skunk-matter through ways in which its olfactory (mal)odours linger in mundane atmospheres, and by doing so, to tease out nuances and long-term violent effects fetid smell has on people and everyday spaces.^1^ Focus on meteorological movements and their everyday bodily sensations, we conclude in the last section, can help in revealing key material functions and atmospheric configurations of olfactory violence, thus allowing a novel take on movements and rhythms of olfactory duration as pivotal for understanding weaponised weathering and the stinky violence it constitutes.

## Violent weathering: Weaponising durations of stink

What ultimately differentiates smelling from other forms of sensing is its close connection to breathing. A living body cannot smell without breathing, inasmuch as breathing ensures olfactory organs, themselves highly adaptive, constantly register small, even subtle differences in motion. The nose sniffs as the body breathes the air that moves. Indeed, breathing is not something the body could voluntarily decide to stop or overcome; it rather signifies the way a sniffing body is constantly dependent on and immersed in its material surroundings – its aerial spheres of atmospheric gaseousness and odorous proximities (on breathing, Joronen, 2023; Kenner, 2021; Nieuwenhuis, 2016; Walker et al., 2020). And yet, in addition to being bound to smell spheres, bodies, like all objects, also produce smell: they spread their odors and mix them with those surrounding them. Smell(ing), in other words, makes bodies utterly atmospheric, something immersed in aerial movements of olfactory matter.^2^

It is such olfactory atmospheres and their immersed materialities that we want to focus on when explicating conditions crucial for how skunk water operates by weaponising (mal)odorousness. As airborne, skunk odours produce volatile spatialities that are constantly in motion: they form varying and immanently shifting degrees of concentration as they blend and dissipate depending on the prevalent weather conditions, such as temperature, humidity, the direction of the wind, and the speed of airflows. Indeed, smell does not only engender spheric proximities, the smell-spheres of ‘sniffing noses’ and ‘fragrant bodies/objects’ mentioned above; smell also moves in the air, spreads through its decentred movements, and reforms through its turbulences. Smell is an aerial matter in motion. It lingers, endures, and wafts in the air. It has a duration that shifts and moves. To think of the materiality of the smell is, in other words, a way of thinking of its lingering *duration in motion*.

How can such duration of moving matter then be weaponised as olfactory atmospheres? Despite the way the skunk smell operates by targeting sniffing bodies immersed in movements of air, skunk water cannot be considered as just another transient thing with a bad smell. Its development is part of a longer genealogy of manufacturing less-than-lethal aerial crowd control tools, which took off after the First World War when tear gas was used, contrary to the ‘1925 Geneva Protocol’ that categorised tear gas as a chemical warfare agent and banned its use in war, against civilians in the US and by the British in their colonies. For the British, the 1919 Punjab massacre in India played a crucial role, albeit it was the Palestinian revolt in 1929 and the upholding demonstrations and strikes in the mid-1930s that were the key events boosting the formal British approval of the use of tear gas for more “humanitarian” policing of the colonies (Feigenbaum, 2017; Legg, 2007). Here already the human body became targeted as a breathing entity captured in aerial environs of toxic clouds that cause various, (mostly) less-than-lethal symptoms (respiratory difficulties, eye and skin irritation, etc.). Albeit skunk water bears resemblance to techniques such as ‘stink bombs’ briefly introduced by the US Army during WWII, and can be further seen as part of the post-Cold War investment in developing a variety of less-than-lethal policing and warfare technologies (Grassiani, 2022; McSorley, 2020; Schmeisser et al., 2013), the particularity of skunk water lies precisely in its non-linear way of weaponising the moving air with a smell that sticks to bodies and objects but also intensifies, spreads and transforms – lingers – according to prevalent weather conditions. Skunk water, in other words, doesn’t simply disperse the mobs; it rather collectively punishes everyone inhabiting the *environments* affected by its sticking, spreading and lingering malodours. Unlike other tools of ‘atmospheric policing’ (Feigenbaum and Kanngieser, 2015), such as tear gas or stun grenades, skunk water *weathers* its violence through atmospheres of lingering movements and tempos of smell.

Important to notice in this regard is, firstly, that the weaponised weathering of smell is conditioned by material movements and meteorological (in)clemencies of the weather, which make the durations of stench always *non-linear* in their movement. This, we argue, is precisely the *modus operandi* the olfactory violence of skunk water operates through: by weathering the flows of air with a smell that moves and lingers, and by so affecting the breathing/smelling bodies exposed to its stench. Such weathering is, albeit always operating through the turbulences of weather, a modality of violence contaminating aerial spheres through the movement of matter. Certainly, the spheres of stink will get dissolved in due course, but this is precisely how such weaponisation operates through a lingering and moving duration of a smell. Indeed, as several scholars have suggested, the chaotic and ungovernable conditions are not simply the opposite of, or mere rhetoric tools for justifying means of governing, but an internal part of how governing works (eg., Joronen and Griffiths, 2022; Kotef and Amir, 2011). Whether this means ‘structured chaos’ (Weizman, 2007), governmentalisation of unpredictability, opaqueness or uncertainly (Berda, 2017; Griffiths and Joronen, 2021), or a logic of governing that works through neglectance, abandonment and absences (Davies et al., 2017; Ramadan and Fregonese 2017; Rose, 2014; Squire, 2015), the recognition of non-linearity *as* governing can serve as a way for thinking ungovernable aspects, as Joronen and Griffiths (2022: 129) conclude, ‘less as a problem to resolve’ and more as something ‘instrumentalised to the ends of governing’. Similarly, meteorological conditions and turbulences of the weather, with all their movements, dissolvement, diffusions, turbulences, and unpredictable declinations, become incorporated into ways in which olfactory violence operates as a moving duration of a stinky matter.

Secondly, while weathering the air with a fetid smell, skunk water also contaminates bodies and the environs it hits and lands on. Such olfactory contamination, we argue, underlines how the weathering of skunk water operates through continuously moving material processes that, on the one hand, contaminate bodies and objects through the air, while on the other, foul the air through the so contaminated bodies and objects. Sniffing bodies smell weathered stink as much as they are forced to become, as wetted bodies, transmitters of sticky skunk smell. Interestingly, this speaks further of the way weathering is a way of weaponising, not merely the aerial, but the *immersion* of breathing and sniffing bodies to their aerial environs. Important to notice here is that the skunk water, when drying and getting stuck to its targets, can reactivate its fetid smell under particular weather conditions, which can happen, for instance, when the stench becomes intensified by rain/humidity, sun/heat or wind speed/direction, all reacting differently with various material forms of skunk (the cloud, the water, the droplets, the dried powder). Duration of skunk water smell, we thus argue, is not only centred on the breathing body with a sniffing nose but importantly operates – lingers and evolves – through decentring flows of air. In other words: atmospheric violence materialises when the skunk smell floats in the air, or when the smell spreads beyond its sites of use through moving bodies, solids, or a steady breeze, but also, when being reactivated or intensified by changing weather conditions. Crucially, such variations do not make olfactory weathering a random process but rather provide a view to non-linear and transformative movements that extend violent weathering in spatial and temporal terms. Duration of smell, in short, contains varying tempos and rhythms of spatialisation. These variations, we explicate next, are a crucial part of how the olfactory violence of skunk water weathering works.

## Stinky matter in motion: Meteorological fluidities and lingering proximities

In his book, *The Life of Plants: A Metaphysics of Mixture*, Emanuele Coccia (2019) approaches the materiality of the air from a surprisingly earth-focused aspect: as an atmospheric continuation of earth's fluid water-worlds. The change between the late Cambrian and early Ordovician period, when life moved from seas to gaseous atmosphere produced by the first cyanobacteria capable of photosynthesis, and thus, of oxygen accumulation, is not, Coccia argues, a radical change overcoming the fluid water milieus of life, but a *transformation* of material fluidity from a watery to an aerial one. The movement of life from the sea to earth was, ultimately, not a conquest of the earth, but a conquest of the air through a fundamental transformation of the fluid sphere of living. Importantly, Coccia underlines, such a turn from the oceanic hydrosphere to a gaseous atmosphere should not be understood as a local event, but as a global symbiogenesis, where breathing organisms became immersed in, and ultimately dependent on both: the fluid gaseousness of the atmosphere, and the life of other living beings (plants) that constantly re-produce this gaseous sphere through photosynthesis.

Without getting into too many details of Coccia's discussion of plants and their life-producing ‘metaphysics of mixture’, it is important to notice that the work provides a geohistorical perspective on why fluidity should not be seen as external to but rather as constitutive for the materiality of atmospheric violence (on fluidity in geography, see Anderson and Wylie, 2009; Cresswell and Martin, 2012; Steinberg and Peters, 2015). Such understanding of aerial fluidity, we argue, is crucial for explicating the roles that moving, transforming, lingering, and dissolving materialities have in forming the olfactory violence of skunk water. The breathing body is embedded in atmospheric fluidities of air like a fish in the water. The olfactionl, we however argue, can never form a global atmosphere. It rather works through more situated *weatherings* closely related to local turbulences of air, and to ways in which skunk water takes different material forms and spreads through moving bodies/objects. Indeed, when shot from a water cannon, the skunk water first spreads in the turbulences of the air as a cloud of mist before landing on bodies and objects it wets, eventually drying on them and becoming part of the bodies/objects that further transmit and spread its stink to the (moving) air. The olfactory materiality of the skunk water is, in other words, *meteorological*: it is related to material morphoses (from liquid and clouds to droplets and dried powder), prevalent weather conditions (reactivating/catalysing/dissolving the smell) and material kinetics (of contaminated bodies and objects) that constitute odorous durations through a moving matter with a stinking sphere.

To offer a better grasp on what we mean by the meteorological aspect of weathering, we want to shortly turn to Michel Serres’ discussion of *meteora* in *The Birth of Physics* (2018) and *Genesis* (1995) (on Serres in geography, see Hayes, 2022; McCormack, 2014; Ojani, 2022)*.* As a meteorological composition of clouds, rain, waterspouts, hailstorms, wind, thunder, cyclones, and so on, *meteora* names precisely what turns the focus from the globality of earth's atmosphere (Coccia) to the event-like proximities of accidental ‘here and now’ – to the ‘clemency and inclemency of the weather’, as Heidegger (2001:147) aptly put it. This aerial proximity of the weather, Serres (2018: 89) interestingly starts his discussion in the *Birth of Physics* (explicitly focusing on the Roman-era atomic thinker Lucretius), has found a repressed form in modern physics*: meteora* is precisely what the closed and controlled ‘laboratory’ systems needed to exclude from their view. What escapes the abstractions crucial for these closed systems – what is left undervalued to the extent that the ‘lightning, rain and clouds’ were found ‘incomprehensible’ – is what belongs, Serres (2018:109) writes, to ‘the peasant or the sailor, the agronomist, geographer, the oceanographer’. Indeed, *meteora* names what remains the chaos-driven ‘here and now’, the *geographical* proximity of material movement that might engender durations and homeorhetic equilibriums, but which flows remain turbulent, always returning to the disorient weathering(s) and aerial motions. *Meteora*, thus, does not simply refer to chaos and disorder but rather forms a narrow space between the *turba* and the *turbo*, the former signifying the disorder and the *tumult* –turbulence – and the latter a particular *form* of movement – a vortex. In other words, *meteora* points to a movement of matter, where meteorological turbulences appear and may temporarily retain a moving order – a duration or equilibrium – but where they eventually become dissolved, spread, and undone.

The focus on meteorology, we argue, helps in underlining olfactory materialities in their lingering durations and, perhaps more importantly, in their movement. Forms in movement are not states, they are *rhythms*: moving forms, like vortexes, spin and take shape, but still contain shaking equilibriums when appearing. Think, for instance, the movement of a ‘spinning top’: it remains stable as long as it moves, but also constantly balances while its axis leans, and its location oscillates (Serres 2018: 49–50). Yet, no motion maintains its tempo forever. Vortexes break down, movement gets disrupted, flows become fragile, and conditions wear out, leak, seep, deviate and dissolve. They spread and move in manifold directions. Indeed, as Serres (2018: 91) bluntly points out, ‘effusion is a diffusion’: the fetid smell of skunk water alike, when effusing, is always ultimately its diffusion, an olfactory flow spreading through the fluctuations of air. The focus on the meteorology of the olfactory violence (of skunk) is thus an aim to comprehend its lingering movement in turbulences of the weather. What we want to argue here is precisely that such meteorological focus on weathering can help in highlighting how the violence of olfactory materialities operates as a *duration in motion*: through diffusions, dissipations, reactivations, metamorphoses, equilibriums, deviations, and so on that make the sticky smell of skunk water to abide, spread, and linger through movement. This, we argue, can further highlight the *volumetric* aspect of aerial violence, not simply as vertical (Weizman, 2007) or defined by angles, inclines, and directions (Billé, 2020; Elden, 2013), but through the motion of matter that reforms the volumetric duration of olfaction in a processual, non-linear and erratic manner. Such moving and lingering smell might not always take the (spatial) form of a vortex (see Ghantous, 2023; Woodward and Jones, 2021) but can nevertheless retain an order or a form, for instance, when staying in motion through fluctuating tempos that engender enduring and latent (mal)odorousness (the latter being the case when the smell becomes reactivated by changing weather conditions). As a moving matter, the skunk water stench is not simply transient, but a weaponisation designed to stick and endure through movement.

As the discussion above shows, we approach weaponised weathering as a duration of smell formed through situated meteorologies of atmospheric materiality, which, despite the turbulence, contains certain forms of movement – certain lingering tempos and space-making durations. Indeed, while the atmospheric has, as McCormack (2014) reminds, meteorological and affective aspects, when the focus is turned to olfactory materiality, the meteorological aspect, we add, needs to be further seen to contain both, the moving rhythms and the lingering proximities. Importantly then, as much as weathering is weaponised through rhythms that transform and move the lingering smell, the spheres of smell abide and recompose by moving along the tempos of the weather. Olfactory duration, in short, is a *lingering rhythm* of a *moving matter*: weathering temporalises moving duration(s) as much as it spatialises recomposing nearness(es) of smell.^3^

## Stench atmospheres in Palestine: Skunk water as weathering technology

In 2003, a public commission in Israel, named ‘the Or Commission’, released its recommendations regarding the mass uprising among Palestinian citizens of Israel that the Israeli police quelled (as part of the wider uprising of Second Intifada that broke out in September 2000) with extreme violence resulting in numerous injuries and the killing of 12 citizens. Commission's recommendations explicitly criticised Israeli Police's handling of the events, stating that live ammunition and snipers should not be deployed to disperse protests. Instead, the Commission suggested the Police to use and develop alternative, ‘non-lethal’ crowd control technologies (Or Commission, 2003). In the spirit of this recommendation, a year later, in 2004, the Israeli Police developed, together with the Israeli company Odortec Ltd, what has become known as the skunk water: a less-than-lethal crowd control tool that uses extremely foul-smelling liquid as a mean to disperse rioting masses. According to its ‘Material and Safety Data Sheet’ (MSDS), the basic components of skunk water consist of yeasts, water, and sodium bicarbonate (baking soda) – though according to other sources, some ingredients were kept in secret – so that in the manufacturing process the ‘yeasts synthesize acids’, thus ‘causing a heavy odor’ (MSDS, 2004; see also Davies, 2008). Albeit acknowledging some of the potential harms – skin irritation, redness and pain in the eyes, and abdominal pain and breathing difficulties when ingested – the MSDS further classifies skunk water as non-toxic and environmental-friendly, the Head of the Technological Development Department of the Israeli Police, David Ben Harosh, even announcing it safe enough to drink (Ben-Simhon, 2008).

**Figure 1.**
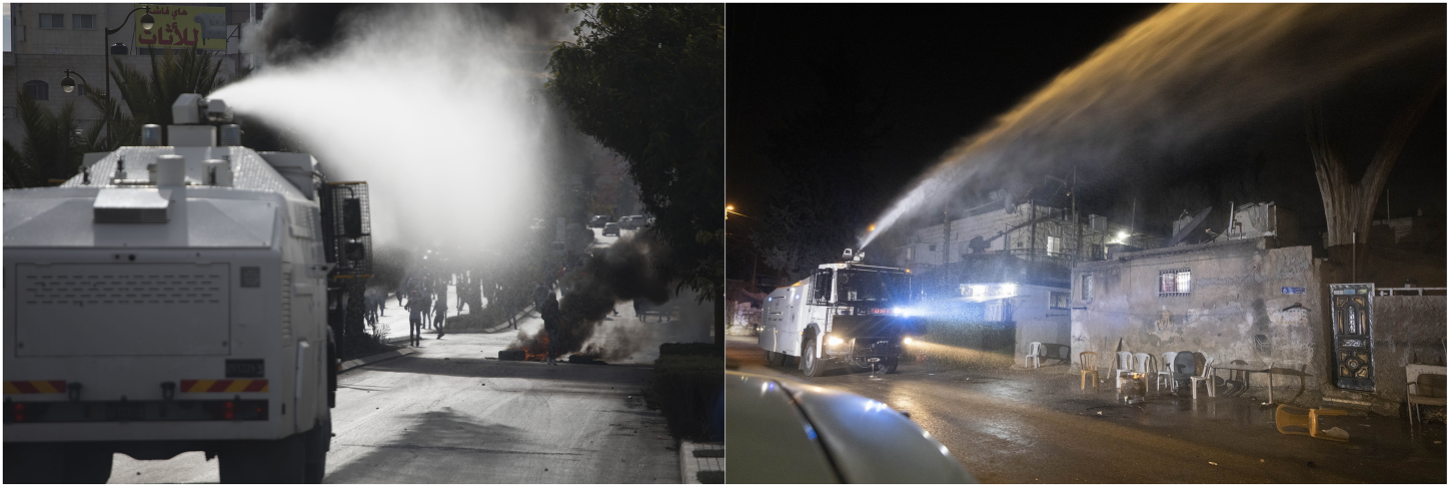
Weathering the air with ‘Kharrarah' in East Jerusalem. Photos by ActiveStills.

Four years later, in 2008, Israeli border police used skunk water for the first time to quell the weekly protests against the Separation Wall in the Palestinian village of Ni’lin, located in the Northern West Bank (Davies, 2008). At that time, skunk water was sprayed from containers carried on the backs of the Israeli forces, a method that was abandoned thereafter due to the wetting of operators (B’Tselem, 2013). Since then, skunk water has been fired primarily from a specially designed armoured truck (manufactured by Beit Alfa Technologies) – the Kharrarah – equipped with a water cannon capable of spraying the water up to a distance of 40 m (see Figure 1). Recently, the Israeli military has also deployed ‘Shoko drones’ that have dropped skunk water on protesters, for instance during the 2018 “Great March of Return” in Gaza, while there have also been reports of Gazan fishermen being sprayed with skunk water from Israeli gunboats (eg., Mahmoud and Shehada, 2019; QNN, 2022; UN HRC, 2019). In 2014, skunk water crossed the Green Line for the first time, when used in the city of Taybeh against Palestinian citizens of Israel protesting against home demolitions ([Bibr bibr1-25148486241226923][Bibr bibr1-25148486241226923]). Since then, skunk water has been used intermittently against Palestinian citizens of Israel, but also later on, in demonstrations held by marginalised groups within Israeli society, primarily the anti-Zionist ultraorthodox Jewish community in Jerusalem known as the *Edah haCharedit* (‘Congregation of God-Fearers’).^4^ The May 2021 escalation (the ‘Unity Intifada’) eventually bore witness to an almost daily use against Palestinians both in the occupied territories as well as inside Israel ([Bibr bibr2-25148486241226923][Bibr bibr2-25148486241226923]; see also Ziv 2023).

### Splashing and misting: clouds, solids, moving bodies

As the original 2014 Police manual (for regulating skunk water operations) states, the liquid is meant for ‘open environment’ use only Figure 1. The manual prohibits the use in closed spaces, further underlining skunk water should not be used in religious sites, or against pregnant women, elderly, infants and subjects positioned on elevated places (which may result in injury if they fall) (Israel Police, 2014: 4). Interestingly, as the section ‘modes of operation’ clarifies, skunk water spraying is framed as a ‘wetting method’ that aims at ‘indirect splashing’. According to the manual, it should be aimed above protestors’ heads (or alternatively on their legs) in a swirling movement, so that the skunk liquid is splintered ‘sideways – back and forth – in a way that resembles the movement of a sprinkler’ and thus produces ‘a rain-like effect’. Such operation, the manual further instructs, should be carried out ‘along wind direction’, otherwise the operators themselves could be exposed to the skunk clouds (Israel Police, 2014: 4–5). After emphasising the tool needs to be deployed only at ‘level C’ protests (eg., in violent protests)^5^ and in close coordination with the use of other crowd control tools (tear gas canisters, rubber bullets etc.), the manual points out the order through which the spraying should be carried out: after an announced warning, one short shot over the protestors should be made, and the spraying ceased to examine reactions and effects. If the desired goals were not achieved, the operation should be repeated in the same order.

Beyond the formal guidelines, however, the skunk water spraying in the occupied Palestinian territories (including the illegally annexed East Jerusalem on which the new instructions *should* apply, see footnote 4) reveals a rather different reality of wetting colonised bodies, homes and entire neighbourhoods/villages. As numerous Human Rights NGOs and media reports (eg. B’Tselem, 2013; Ziv, 2023), publicly available video materials (eg. Matar, 2014), testimonies of protestors and residents (eg. Jundi, 2021), and the materials we collected reveal (see footnote 1), the only accomplished aspects of the skunk water manual are, indeed, those related to weathering: the ‘sprinkling’, the ‘wetting’, ‘the splashing’, the ‘wind’, and the ‘rain-effect’. Otherwise, the instructions have been systematically ignored (or intentionally violated), when homes, religious sites, schools, vegetable and clothes shops, gas stations, hotels, backyards, gardens, bypassers, and even entire villages, have been sprayed. As the words of East Jerusalemite shopkeeper, Suleiman, highlight:

It is not that they [Boarder Police] use skunk water to disperse big demonstrations, or whatever; it is enough to have a couple of kids around, *they will drive after them and start spraying the whole area*. They target the shops, which has massive effects, as people lose their sources of income. People who have clothing stores cannot sell clothes anymore, fruits are being thrown away, shops need to be closed for days, and so on [our emphasis]

He then added a telling example:

The other day there was a truck carrying tons of bananas, when the *Kharrarah* started spraying its stinky water all over it. The driver could see all the bananas carried on the truck, he made it on purpose […] This is clearly intentional, they have double aims: one, to make us [shop owners] turn against the demonstrators, which we will never do, as Jerusalem comes first; two, they want to empty the area from shops, and make us shut down and leave.

**Figure 2.**
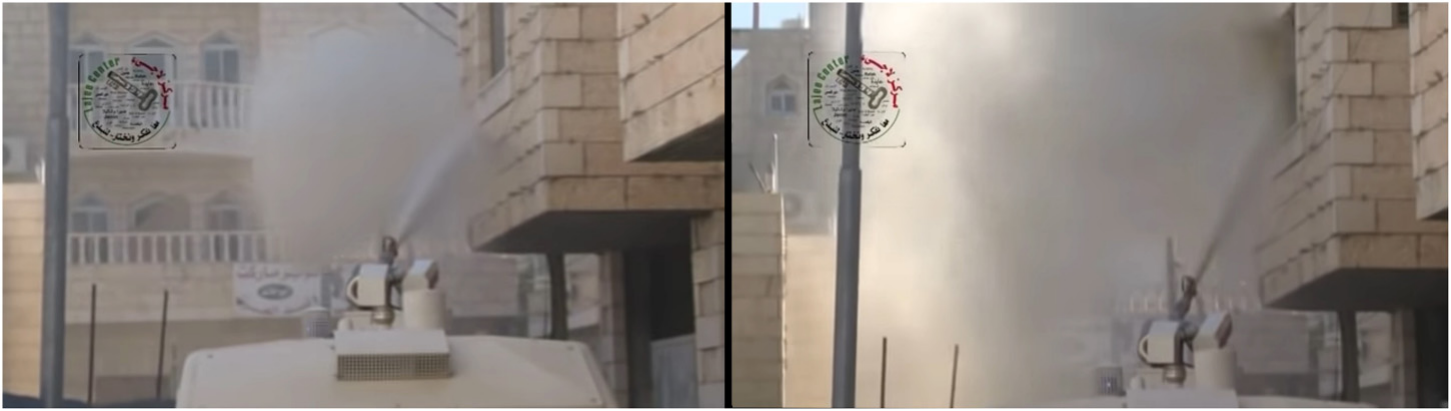
Spraying the façade and inside of houses at West Bank refugee camp. Stills from a video footage by Mohammad Alazza.

As the words of Suleiman underline, skunk water use is not restricted to quelling violent protests, but contains several functions, ranging from collective punishment and expulsion to humiliation, disciplining and inflicting of financial losses. This was further elaborated by Manal from Aida camp, who painstakingly went through the events of her family home being sprayed, from a few metres distance, with *Kharrarah* (See Figure 2 below):

The high pressure of water broke the windows and the water got into the house. But that's not the issue, the issue is that when it happened, we couldn't sleep in the house for several days because of the stink.

In addition to the splashing of homes, windows, shops, and banana cargos with direct high-pressure water, skunk water is often sprayed, as Jerusalem-based student Rawan explained, ‘randomly to many directions’ from a ‘moving cannon that revolves around itself’. Such ‘rain-effect’ engenders a skunk cloud that indiscriminately gets stuck at everything it hits – shoes, walls, balconies, windows, as our interviewees further listed.

Skunk water use, indeed, weathers the air with its fluid stench. Its smelly clouds move in the air as a mist, land and stick on objects, spaces and bodies, and so force them to become transmitters of its repulsive malodour. Crucially, one doesn’t need to get all wetted by the skunk water. ‘Only one drop is sufficient to make the smell stick’, as Rawan asserted, thus underlining what almost all the interviewees pointed out – namely, that the skunk water is highly contagious and contaminates everything in touch with it. Mohammad confirmed this when going through aspects that separate skunk water from rubber bullets (that target people individually) and tear gasses (that dissolve in the air relatively quickly):

But the skunk water, once it hits you, it stays two or three weeks, even a month, so it has a long-lasting effect and that's very frustrating cause you smell it all the time. Even after it wears off, you can still smell it.

A journalist, Amir (from Aida camp), expanded:

There's no need to get fully soaked, just a little bit of the skunk water gets on you, that's enough, my vest, my hat, my shoes, my clothes, even my camera got covered with that disgusting smell. It got to a point where I said I’m leaving my camera behind because I couldn't hold it – it stank, I tried to clean it using pure alcohol; some people suggested using citric acid and tomato juice; but nothing helped.

As the quotes highlight, the skunk smell abides in bodies, objects and spaces it hits to the extent that after several showers and the use of various ingredients for cleaning oneself (and the objects/surfaces), the stink lasted for days, even for weeks – in some cases for months. The effects of abiding smell can thus resemble torture: one is not only forced to continuously smell the repulsive stench until it wears off but most importantly can also keep smelling it after the stench has dissipated.

As the skunk spray lands and dries up, it becomes a fetid solid. Its smell sticks to sprayed bodies/objects/surfaces from which it continues to expand its olfactory weathering. This was further evidenced by Amir, who explained how even an ‘accidental touch on a wall contaminated by skunk water’ can make ‘the smell stick to your hands’, and consequently, spread beyond the original sites of use. Manal, from Aida camp, further opened up the vicious circle present here: ‘to be honest’, she started, ‘it's very frustrating because the smell stays in the streets, inside the house, in the carpets, in the walls; every time you go outside it gets on your shoes.’ Indeed, the fetid stink does not only remain captured to sprayed skunk clouds; as soluble matter, it transforms from water to clouds, from clouds to droplets and puddles, and eventually, to a dried powder stuck on walls, bodies, cameras, shoes, clothes and so on, through which it further spreads its malodours. Importantly, it is in all these *morphoses* – liquid, mist, drops, dried powder – that the skunk water stench *moves* with what it contaminates, some of the targets, such as by-passers shoes and car tires, not even being directly hit by the skunk water cloud. As Jalal and Mahmoud, two shop owners from East Jerusalem, concluded:

Last Saturday they sprayed the whole ground here, and once you walk there, that's it, your shoes are done, the smell doesn’t go away, and you carry the smell along with you. The shoes I washed, but the smell is still there, and it travels with you wherever you go – to the house, to the car. You carry the smell with you and spread it wherever you go. Your family is also affected, not only you.

Moving bodies and objects, as the quotes above exemplify, play an important role in weathering the olfactory stench. The stench moves along the sprayed bodies, and the clothes they wear, but also through objects it contaminates, such as cameras and car tires. Contaminated bodies and objects, when in touch with different forms of skunk water, function as moving solids through which the skunk particles and the smell spheres they produce travel outside the original sites of spraying.

#### Floating atmospheres, reactivating weather

Skunk water, the discussion so far has shown, creates moving proximities of smell that weather the air, not only when sprayed as skunk clouds, but also when emanating from bodies, objects and spaces, which the skunk water has landed on, dried into, and/or moved along with. Skunk water, in other words, *weathers the air*, by moving from the air to bodies/objects and from bodies/objects to the air, in the process taking gaseous, liquid, and solid forms. Importantly, skunk stench thus does not spread only through watery forms but also through aerial and solid ones. It forms a spatial movement, an atmospheric materiality, that hovers, lingers, emanates, reactivates and intensifies. As Rawan opens up the olfactory atmospheres so engendered:

“The smell stays with you, and it stays in *the space too*. A while ago, and for many days, if you walked through Bab al-’Amood [Damascus Gate; Jerusalem old city] all the way to Sheikh Jarrah [an East Jerusalem neighbourhood], all you could smell was shit.’ [our emphasis]

As the words of Rawan highlight, skunk stench can linger in the air and spread to different neighbourhoods. Lama, from Sheikh Jarrah, described this at length in the aftermath of May 2021 spraying in front of ‘Bab al-‘Amood’ (Damascus Gate), when the ‘smell was particularly strong’, reaching all the way ‘to the church of Holy Sepulchre’ on the other side of the old city. At *Laylat al-Qadr* (‘Night of Power’), the holiest night of the Ramadan month, Lama elaborated, ‘people celebrated and went shopping in the old city’, and ‘because people were passing through sprayed areas with puddles of skunk water’, the deeper they walked down from the Damascus Gate to the old city, more strongly the smell ‘entered into the deep arteries of the old city’, eventually wafting and lingering in – weathering – its narrow alleys and labyrinthine avenues. Indeed, the smell can hover and spread in the air to the extent that the nearby shops need to ‘close the doors temporarily’, even if not directly hit by the skunk water, as several East Jerusalemite shopkeepers explained. In addition, the skunk smell can also saturate in the ‘crowded and narrow alleys’ of refugee camps or old city centres, as Amir from Aida camp added.

The smell, it seems, can weather the air by floating in it, even to the extent of weathering areas not directly sprayed with the skunk water. The stench spreads through people's shoes and belongings, as much as it spreads through the air, especially if the weather conditions are favourable. Indeed, weather conditions play an important role in constituting the aerial movements and durations of olfactory weathering. They can intensify and extend as much as diffuse and reactivate the skunk smell, thus constituting the aerial materialities through which the olfaction moves and abides. As Lama describes the role of weather conditions:

“It [the skunk] spreads and sticks everywhere and in everything: your home, your car, your private space, your public space, your body. It lasts, and *if the next day is hot, it ferments and it gets worse*.” [our emphasis]

Hot weather conditions were the key element several interviewees mentioned as capable of prolonging the lingering of skunk smell. Together with increasing levels of humidity and lack of wind, the heat can significantly intensify the stench: humidity in the air can capture odour molecules, thus making them spread farther and linger longer, while the warm air, in addition to being capable of binding more moisture to itself and so of trapping more odour, can intensify the smell by increasing volatilisation and the number of airborne odour particles, but also by increasing movement and uplift of odour molecules. Together with relatively stable wind conditions, these weather elements can make the smell, literally, float in the air. As one of our interviewees aptly described:

After they spray one area, the smell travels and expands to other areas, especially in hot weather. It becomes hard to identify where it comes from, it fills the entire space as an invisible cloud.

Skunk water, the above shows, weathers its repulsive stench. It fills the air, moves as an ‘invisible cloud’, and engenders olfactory atmospheres that abide in movement. Though containing olfactory proximities and volumetric durations, skunk weathering never operates as a static sphere of olfaction; it rather moves and recomposes itself as a moving and lingering duration. Rather than simply dissolving the smell in the air, the weather conditions intensify, expand, reactivate, and prolong its malodour. Meteorological conditions, in other words, are not external forces working against the skunk water weathering but constitute those moving materialities of smell through which the skunk stench operates. As the skunk water weathers durations that linger in the air, it simultaneously becomes weathered by certain meteorological conditions, and the aerial movements of matter they foment. The lingering stink persists precisely because certain weather conditions operate as material means for weaponising the effluvium.

It is crucial to add that the prolonging of smell is not necessarily a linear process of intensification, but also takes place through what we call a process of *reactivation*. As Amir explains:

The problem is that, when you try to clean it [the skunk] with water, the stench becomes even worse, and the heat from the sun and humidity increase it too, and to add to this, with the tear gas thrown in the area, it all gets mixed up.

Certainly, here the various aspects of weathering – the heat, the humidity, the air pressure, and the movement of air – come comprehensively together as part of the way skunk stench abides, lingers, and spreads as a moving matter. And yet, as Amir emphasised above, it is water that reactivates and re-wets the stink. This was elaborated in detail by Manal (from the Aida camp), whose family home was, as explained above (see Figure 2), targeted from a short distance with high-pressure skunk water, to the extent that the pressure broke the windows and the metal covers that protected them, eventually getting inside the living room. As Manal explained:

At that time, it was winter and *every time it rained the smell got worse*. We tried everything you can imagine, but nothing helped, we tried bleach, we tried perfume, nothing helped. The first time our neighbour brought a water tank and cleaned the entire neighbourhood, but it was useless*. Every time we cleaned with water the smell would come back*. [our emphases]

Manal then added an important point:

The problem is that we didn’t put fillings between the stones that cover the house's façade, so *the water got absorbed into the wall*. [our emphasis]

As Figure 2 highlights, Manal's family house's façade wasn’t finished at the time of spraying and thus did not have a mortal between the tiles. The skunk water, as being shot with high pressure from a short distance, drained between and behind the tiles, eventually drying inside the wall. The use of water, as Manal explained, only made the situation worse. As dried between the tiles, the smell kept reactivating when in touch with water (coming from rain or cleaning), thus constituting another, more transformative rhythm of olfactory duration.

What is crucial here is not only the evidently intentional spraying of people's homes but the fact that the skunk water – as stuck between the tiles – dried and eventually got reactivated by rain and cleaning with water. Skunk smell thus doesn’t only abide through morphoses from water to droplets and from droplets to dried powder; it also intensifies through re-wetting conditions – rain, cleaning, humidity – that can further re-activate the powder and its stench. Such reactivations thus provide an additional form of olfactory temporality, a *rhythm* through which the duration of stink abides. Along with the intensifications catalysed by heat and humidity, such changes engender tempos that, instead of spreading and dissolving the smell linearly, produce olfactory durations through the rhythms of changing weather conditions.

As the discussion above shows, the repulsive smell of skunk water lingers and spreads – has its durations and meteorological proximities – through several forms of movement: (a) through material morphoses, where the sprayed water transforms to misty clouds, the misty clouds to droplets and puddles, droplets and puddles to dried powder, and the dried powder to gaseous and re-wetted stink; (b) through material kinetics of fetid solids; namely, the moving bodies and objects contaminated by skunk smell; and (c) through reactivating and intensifying weather conditions – the rain, the heat, the humidity, the wind. All these conditions engender the abiding, yet dispersing and re-intensifying stench in motion that, when temporalising (as duration) and spatialising (as lingering proximity), constitute the violent olfactory weathering of entire neighbourhoods and communities: namely, the weaponisation of moving matter with a stinking sphere.

## Conclusions

In this paper, we have elaborated the two sides of what we call violent weathering – the meteorological and the weaponised – as they come together in atmospheric materialisations of lingering skunk water stench. Such weathering, we argue, operates through atmospheric proximities and durations that move – intensify, transform, reactivate, spread, and waft – in the air due to certain weather conditions, ranging from temperature and humidity to air pressure, rain and wind direction, as well as through the morphoses and movements of stinky matter. Olfactory materialities, in other words, are in motion, forming tempos and fluid atmospheres of stench. Such temporalising duration and spatialising movement of smelly matter might need to operate through meteorological turbulence and changing weather conditions, but this is precisely, we argue, how the skunk water operates when exposing sniffing and breathing bodies, even entire communities, to material atmospheres of stink. The stench, designed to stick, can linger in the streets of entire neighbourhoods or, as an invisible could, move into and fill neighbouring areas, as much as it can spread through movement of solid forms – shoes, cameras, car tires, fouled bodies, and more – so turned to transmitters of its malodours. Moreover, skunk water can weather the air by transforming from water to mist, from mist to drops and puddles, from drops and puddles to dried powder, and from dried powder to re-wetted and re-activated smell. Skunk water, in short, is not simply a sprayed cloud of water: it is a weaponisation of moving smell that targets the *environments* that bodies inhabit. Its transforming stench (liquid, solid, and gaseous) operates as a duration that recomposes and lingers in motion.

Such moving duration, we want to conclude, is never a linear process of smell dissipating through turbulent fluctuations and fluidities of moving air. Such duration rather contains tempos that, through a combination of (a) bodily kinetics, (b) recomposing material morphoses and (c) re-activating meteorological intensifications, constitute ways in which weaponised weathering spatialises itself as a moving, lingering and rhythmic atmosphere of smell. This, we argue firstly, underlines the importance of approaching the materiality of violence *in motion* (eg. Ghantous and Joronen, 2022), our discussion hence offering a kinetic addition to debates around the atmospheric and infrastructural materialities (eg. Fregonese, 2020; Pugliese, 2020; Salamanca and Silver, 2022), post/more-than/less-than-human geographies of violence (eg. Braverman, 2021; Delso, 2018; Philo, 2017; 2021), and the volumetric nature of power (eg. Adey, 2013; Elden, 2013; Weizman, 2007). Above all, it speaks of the non-linearity of violence and weaponisation of ungovernable conditions as material means of governing, subjugation and colonial violence (Joronen and Griffiths 2022; Kotef and Amir, 2011; Weizman, 2007).

Secondly, the focus on the materiality of the skunk water aims to describe the operations of yet another repulsive *colonial tool* aiming to weather the colonised population, and their everyday material/aerial environs (see Griffiths, 2022; Povinelli, 2016; Stamatopoulus-Robbins, 2022). Indeed, the use of the tool should be seen as part of a longer colonial history of producing the ‘filthy’ and ‘smelly Others’ (and the various violent attempts to then ‘sanitise’ them) not only through ‘racist representations’ (eg. Classen et al., 1994; Hsu, 2020) but importantly, we argue, through the material marking – namely, through the olfactory weathering of bodies, neighbourhoods, and atmospheres with a stench designed to stick. Such a repulsive weathering tool thus speaks further of the negativity of material atmospheres (Joronen, 2023), particularly of their way of engendering effluvium and fetid olfactory violence against colonised bodies, objects, and mundane spaces.

Finally, while we have opened up various violent effects of skunk water use, more thorough work is still needed to explicate the wider social, political, economic, environmental, affective and bodily effects of olfactory policing and weaponisation of smell. While the skunk water weathering violently entraps people to stench clouds, also the lingering duration of smell causes various effects, ranging from social exclusion and nausea to othering of targeted communities, financial losses, collective punishment, and contamination of everyday spaces of living. Indeed, as much as weathering is a material process, it also constitutes various *affective atmospheres*, for instance when humiliating or slowly wearing down those who dwell in sites of spraying (Harker, 2020; Joronen, 2021). And yet, as the repeated use of the Arab word Kharrarah – the ‘shit-spreader’ – among our Palestinian interlocutors exemplifies, on many occasions, people relied on *humour* and laughter as means to downplay the violent effects of skunk smell – the humiliation, the frustration, the embarrassment, and so on – while also describing various other ways of resisting the skunk machinery. Such counteracts and -attunements further highlight the importance of recognising the role of negative materialities, not only in building (colonial) worlds but also in ending them (Dekeyser, 2022), as recalled in various recent works in geography focusing on the unmaking of oppressive worlds (eg. Chandler and Pugh, 2022; Griffiths and Joronen 2023; Noxolo, 2022; Sidaway, 2022). As breathing and sniffing entities, Palestinian bodies might be immersed in their aerial environs and gaseous atmospheres, even forced to become transmitters of weaponised malodours, yet without being ever completely subjugated to mere sufferers of stench (as unpleasant as it is) or tamed with material weathering (as violent and repulsive as they can be) (see Hammami, 2015; Shalhoub-Kevorkian, 2016). The atmospheric immersions of those targeted with olfactory violence remain fluid and turbulent alike – turmoil(s) always irreducible to atmospheric weaponisations of lingering stink.

## Highlights

- The notion of weathering offers a meteorological and material take on the atmospheric weaponisation of smell.
- Skunk water spraying targets the way breathing bodies are immersed in their environs.
- Malodours are weaponised as a non-linear way of governing meteorological movements and lingering proximities of stench atmospheres.
- Intensifying weather conditions, morphoses of matter, and moving bodies/objects are central for comprehending the atmospheric duration of skunk water as a lingering effluvium in motion.

## Notes

1. Materials were collected in East Jerusalem and the West Bank during four fieldwork trips conducted between May 2019 and June 2021. Methods used include participatory research, interviews with affected people (shop owners, residents of sprayed areas, hotel personnel, journalists, protesters, students going to university) and a collection of various archival materials (newspaper articles, videos, NGO materials, policy papers and skunk water use manuals/instructions).

2. Unlike sonic or visual sensing, which materiality can be mapped through wavelength, olfactory organs constantly adapt to manifold molecular features with changing mixtures and concentrations that not only stimulate nerve impulses received at brain's limbic system responsible for memories and emotions but which reception is also related to body's life-worlds, and the cultural, spatial, material, and social histories it carries along (see Barwich, 2020; Cobb 2020).

3. As Serres (1995: 102) notes, in French the word *temps* refers tellingly to both, the time and the weather. It is through the disorderly weathering, that the lengths of duration and ‘the ordered times of clocks, organisms and systems’ are formed.

4. It is worth noting that in 2018, a group of affected Jewish ultraorthodox communities appealed to the Israeli High Court of Justice to prohibit the use of skunk water in residential and crowded areas. The court rejected the appeal on the grounds that the Israeli Police updated its operative manual for skunk water use in a way that demands stricter regulations when used in residential and crowded areas (see HCJ 5882/18). The Police manual, however, is valid only for skunk water use in Jerusalem, whereas the West Bank falls under the responsibility of the Israeli Military. Despite the fact that within Israeli society skunk water is used less frequently, less intensively, for different purposes and by following different regulations – also often raising a wave of public condemnation – such use importantly speaks of the way colonial techniques boomerang ‘back home’.

5. For more information on ‘level C’ disturbances to public order, see Article 154 of the Israel Penal Code.
